# Critical appraisal of the prognostic value of presepsin in sepsis: methodological and interpretive concerns

**DOI:** 10.1590/1806-9282.20250733

**Published:** 2025-10-27

**Authors:** Yanlin Wu, Ruyi Zheng, Yuxin Xie, Aili Fang

**Affiliations:** 1 Hangzhou Normal University, School of Clinical Medicine – Hangzhou, China.; 2 The Third Affiliated Hospital of Wenzhou Medical University, Ruian People's Hospital – Wenzhou, China.

Dear Editor,

I read with great interest the recent article by Uzun et al. titled "Presepsin is a biomarker that can predict mortality in sepsis patients."^1^ This study systematically analyzed presepsin levels in critically ill sepsis patients and reported relatively high sensitivity and specificity for mortality prediction (area under the curve [AUC]=0.819). Moreover, it proposed a specific cutoff value (612.70 pg/mL), providing valuable single-center data supporting the quantitative use of presepsin in the ­clinical prognostic assessment of sepsis. Nevertheless, I would like to respectfully raise several methodological and interpretative concerns that may affect the reliability and ­clinical ­applicability of the findings.

First, insufficient sample size and limited statistical power. The survival group included only 30 patients (34.48%) compared to 57 patients in the non-survival group, leading to a notable imbalance in sample size. Such a small sample likely compromises statistical power; for example, the receiver ­operating characteristic (ROC) curve analysis reported sensitivity (73.7%) and specificity (73.3%) without providing corresponding confidence intervals (CIs). Furthermore, the extremely high odds ratio (OR) in the binary logistic regression (OR 81.836, 95%CI 8.436–793.867) strongly suggests potential overfitting of the model. The wide CIs further undermine the credibility of the results, indicating that larger sample sizes are needed for validation. In addition, the cutoff value and model performance were derived solely from single-center data, lacking external cohort validation. Considering the high heterogeneity among sepsis patients, especially the regional specificity of pathogen distribution in areas such as Samsun, multi-center studies are urgently needed to avoid geographic bias and enhance the ­generalizability and reliability of the results.

Second, questionable clinical utility of the positive predictive value (PPV). Although the AUC indicates good ­discriminative ability, the PPV was only 57.35%, implying that only about half of the patients with presepsin levels above 612.70 pg/mL actually died. This PPV is somewhat low compared to commonly accepted clinical decision thresholds (>0.8) for sepsis prognostic biomarkers, thereby diminishing presepsin's ­utility as an independent predictive marker.

Third, neglect of key confounding factors and subgroup analyses. Important confounding factors influencing presepsin levels, such as age (range: 22–97 years, mean: 72.33±12.51), comorbidities, and treatment regimens, were neither controlled nor stratified. Age-associated immunosenescence in elderly sepsis patients may affect biomarker expression^2^; ­however, no age-stratified analysis was performed. Additionally, the prognostic value of presepsin in the fungal infection subgroup (10.34% of cases) was not separately evaluated, limiting the generalizability of the conclusions.

Finally, contradiction with existing meta-analyses and ­inadequate discussion. Reference 7 (Molano-Franco et al., 2023)^3^ is a meta-analysis that firmly concluded that single-time-point presepsin measurements lack significant predictive value for mortality. The current study fails to adequately address these contradictory findings, focusing solely on its favorable AUC while neglecting the exploration of heterogeneity sources such as the timing of measurement and patient population ­differences, which raises concerns of selective bias.

In conclusion, although presepsin shows promise as a sepsis biomarker, the aforementioned methodological issues and insufficient discussion may overstate its prognostic value. We recommend larger-scale, rigorously controlled studies and in-depth comparative analyses to further clarify its ­clinical utility.

## Data Availability

The datasets generated and/or analyzed during the current study are available from the corresponding author upon reasonable request.
